# Upregulation of Cdh1 Attenuates Isoflurane-Induced Neuronal Apoptosis and Long-Term Cognitive Impairments in Developing Rats

**DOI:** 10.3389/fncel.2017.00368

**Published:** 2017-11-23

**Authors:** Xuan Li, Kai Wei, Rong Hu, Bo Zhang, Li Li, Li Wan, Chuanhan Zhang, Wenlong Yao

**Affiliations:** ^1^Department of Anesthesiology, Tongji Hospital, Tongji Medical College, Huazhong University of Science and Technology, Wuhan, China; ^2^Department of Physiology, Hubei University of Chinese Medicine, Wuhan, China

**Keywords:** anaphase-promoting complex, Cdh1, isoflurane, apoptosis, cognition

## Abstract

Neonatal exposure to isoflurane can result in neuroapoptosis and persistent cognitive impairments. However, the underlying mechanisms remain elusive. Anaphase-promoting complex/cyclosome (APC/C) and its co-activator Cdh1 are E3 ubiquitin ligases that play important roles in the central nervous system, including in the regulation of neuronal survival, synaptic development, and mammalian learning and memory. However, whether APC/C-Cdh1 is involved in isoflurane-induced neurotoxicity in developing rats remains unclear. In this study, postnatal day-7 (P7) rat pups and primary hippocampal neurons were exposed to 2% isoflurane for 6 h. Terminal deoxynucleotidyl transferase dUTP nick end labeling (TUNEL) staining was used to detect neuronal apoptosis, and the expression of proteins involved in apoptosis (cleaved caspase-3, Bax and Bcl-2) was assessed by western blot. The level of Cdh1 in the hippocampus was downregulated during isoflurane-induced neuroapoptosis. Cdh1-encoding lentivirus was transfected before isoflurane-treatment to increase the level of Cdh1. Our results showed that Cdh1 overexpression by a recombinant Cdh1-encoding lentivirus reduced isoflurane-induced neuronal apoptosis. Moreover, bilateral intra-hippocampal injection with Cdh1-encoding lentivirus attenuated long-term cognitive deficits after exposure to isoflurane in developing rats. Our study indicates that Cdh1 is an important target to prevent isoflurane-induced developmental neurotoxicity.

## Introduction

Many infants and children receive different types of surgery or diagnostic procedure every year. Recent animal and clinical studies have demonstrated extended or multiple exposures to inhaled anesthetics during brain development can induce neuronal apoptosis and increase the risk of cognitive abnormalities during adulthood (Jevtovic-Todorovic et al., 2003; Zhu et al., 2010; DiMaggio et al., 2011; Cheng et al., 2015; Wu et al., 2017). Isoflurane, as a common volatile anesthetic, has been implicated in long-term developmental neurotoxicity and cognitive deficits (Stratmann et al., 2009).

The mechanisms of isoflurane-induced developmental neurotoxicity are probably multifactorial. First, neonatal exposure to isoflurane can result in neuronal apoptosis (Lei et al., 2012), which affects the normal development of neuronal networks, and leads to long-term neurocognitive decline. The underlying molecular mechanisms include the inhibition of N-methyl-aspartate (NMDA) receptor, activation of γ-aminobutyric acid (GABA) receptor (Fredriksson et al., 2007), dysregulation of intracellular calcium homeostasis (Wei et al., 2008), mitochondrial perturbations (Zhang et al., 2012), downregulation of BNDF (Zhang et al., 2014) and neuroinflammatory pathways (Wang et al., 2016). Second, early anesthesia exposure can disturb synapse development and potentially lead to abnormalities in synaptic plasticity (Huang et al., 2016). Moreover, a recent study has demonstrated that anesthetic suppression of postnatal neurogenesis is associated with deficits in hippocampus-dependent learning and memory function (Kang et al., 2017). Notably, the synaptic plasticity and neurogenesis of neurons can be controlled by the ubiquitin-proteasome system (UPS; Zhou et al., 2016). However, whether ubiquitination activity is related to anesthesia-induced neurotoxicity is unknown.

Anaphase-promoting complex/cyclosome (APC/C), an E3 ubiquitin ligase, and its co-activator, Cdh1, are important components of the UPS. In proliferating cells, APC/C-Cdh1 can prevent premature S phase entry through limiting the accumulation of cyclins in G1 (Li and Zhang, 2009). Neurons are post-mitotic cells. Previous studies have demonstrated that Cdh1 is highly expressed in neurons (Gieffers et al., 1999). Further studies have indicated that APC/C-Cdh1 is involved in axonal growth and patterning, neuronal differentiation, neurogenesis and synaptic development in the central nervous system (Almeida, 2012; Zhou et al., 2016). Almeida et al. (2005) found that Cdh1 phosphorylation by cyclin-dependent kinases (Cdks) promotes post-mitotic neurons re-entry into the cell cycle and mediates excitotoxicity in neurodegenerative disease. Additionally, Cdh1 is essential in maintaining the replicative lifespan of neurons and in hippocampus-dependent cognitive functions (Li et al., 2008). Therefore, we hypothesized that APC/C-Cdh1 could be involved in isoflurane-induced neurotoxicity and cognitive deficits in the developing brain.

In this study, we first examined the change in Cdh1 expression and neuronal apoptosis in the hippocampus of developing rats and, *in vitro*, in primary neurons after exposure to 2% isoflurane for 6 h. We then administered a Cdh1-encoding lentivirus into the hippocampus of rat pups and primary cultured neurons. The effect of Cdh1 overexpression on neuronal apoptosis and the impairment of learning and memory ability induced by isoflurane in developing rats were evaluated.

## Materials and Methods

### Animals

All experimental procedures were approved by the Huazhong University of Science and Technology Ethics Committee for Care and Use of Laboratory Animals. Protocols were in accordance with the National Institute of Health Guide for the Care and Use of Laboratory Animals. Postnatal 1- or 7-day Sprague-Dawley rats of both sexes were supplied by Tongji Medical College Experimental Animal Center. They were housed in a temperature-controlled room with a 12-h light/dark-cycle.

### Primary Hippocampal Neuron Culture

Rat hippocampal neurons were derived from newborn (within 24 h) Sprague-Dawley rats as previously described (Beaudoin et al., 2012). Briefly, hippocampal tissues were dissected, gently minced, and trypsinized. The digestion was stopped by adding DMEM/F12 medium containing 10% fetal bovine serum (FBS). Culture plates were coated with 100 mg/mL Poly-L-lysine. Neurons were cultured at a concentration of 5 × 10^5^ cells/mL onto dishes or coverslips. Then, the medium was changed to neurobasal medium containing B27 supplement (Gibco, USA). We added cytosine arabinoside (10 μM) to the medium to arrest the growth of non-neuronal cells on the second day *in vitro*. Half of the cell medium was replaced every 3 days.

### Anesthesia Procedure

The anesthesia protocol was established according to previous reports (Boscolo et al., 2013; Wang et al., 2014) with some modifications. Rat pups at postnatal day 7 (P7) and cultured neurons were placed in a tightly sealed translucent plastic chamber, which was continuously flushed with fresh gas at 37°C. Rat pups at P7 are susceptible to anesthesia-induced developmental neurotoxicity (Yon et al., 2005). We administered 6 h of isoflurane based on previous studies (Sanchez et al., 2011; Boscolo et al., 2013). The concentration of isoflurane was maintained at 2%. The isoflurane group of rat pups inhaled isoflurane in a gas mixture of 30% oxygen (O_2_) and 70% nitrogen (N_2_). For cultured neurons, isoflurane was flushed in a gas consisting 95% O_2_ and 5% CO_2_ on day 7 *in vitro* (7 DIV). A Datex Capnomac Ultima gas analyzer (Datex Ohmeda, USA) was used to monitor the gas flow (2 L/min). The animals in the control group were handled as the isoflurane-treated animals, except that no isoflurane was added. All rat pups were allowed to breathe spontaneously.

To determine the adequacy of ventilation, arterial blood obtained from the left cardiac ventricle was sampled at the end of anesthesia administration for blood gas analysis as previously described (Jevtovic-Todorovic et al., 2003). There were no obvious signs of metabolic or respiratory distress in the isoflurane compared with the control rats (data not shown). Rat pups assigned for Terminal deoxynucleotidyl transferase dUTP nick end labeling (TUNEL) assays were euthanized immediately post-anesthesia. The hippocampi of rats were removed immediately for biochemical studies.

### Lentivirus Treatment

Cdh1-encoding lentivirus vector was constructed in a previous study (Qi et al., 2014). The encoding sequence of the rat Cdh1 gene (NM_001108074.1) was synthesized chemically. Then, it was fused into the pGC-FU vector through genetic recombination. The lentivirus construct expressing green fluorescent protein (GFP) only (pGC-FU-GFP) was used as a control. The lentiviral package used in our experiments was commercially supplied by GeneChem Inc. (China) and previously used in our laboratory (Qi et al., 2014; Lv et al., 2015). The titer of Cdh1-encoding lentivirus was 1.0 × 10^9^ transduction units (TU)/mL. Neurons and animals were divided into four groups: control (Con) group; isoflurane (Iso) group; Iso + Lenti-Cdh1-GFP group, and Iso + Lenti-GFP group.

GFP expression visualized under a fluorescence microscope was used to determine the optimal multiplicity of infection (MOI) and infection duration. Neurons in the Iso + Lenti-Cdh1-GFP group or the Iso + Lenti-GFP group were transfected with the Cdh1-encoding lentivirus or control lentivirus, respectively, for 10 h at an MOI of 10 on 4 DIV. The medium was then replaced with standard culture medium. Isoflurane exposure was performed 72 h after lentivirus transfection. On 7 DIV, all test neurons were exposed to 2% isoflurane for 6 h. All experiments were repeated three times.

For rat pups (*n* = 16 for each group) in the Iso + Lenti-Cdh1-GFP group and the Iso + Lenti-GFP group, lentivirus (1.5 μL) was directly microinjected into the bilateral hippocampus of rats at postnatal day 3 using a 32-gauge needle (Hamilton Co., Reno, NV, USA; microliter no. 701 RN, 10 μL). The experimental protocol has been described previously (Fitting et al., 2006). The set of coordinates used for the bilateral hippocampus were: −2.0 mm anterior to the Bregma, ±1.6 mm lateral to the Bregma and −2.5 mm dorsal from the dura. The injection lasted 2 min, and the needle was withdrawn over 10 min to prevent reflux. The incision was closed with surgical glue. After two injections, the pups were warmed under a heat lamp before being returned to their cages. Isoflurane exposure was then performed 4 days after lentivirus injection. Rat pups assigned for western blot and TUNEL assays were euthanized immediately post-anesthesia, while the remaining rats (*n* = 10 for each group) were placed back to their cages for behavioral tests.

### TUNEL Staining

After euthanasia with pentobarbital injection (100 mg/kg, intraperitoneal), the brains were collected and treated with 4% paraformaldehyde (PFA) in PBS. Neurons were washed with D-Hanks and then fixed with PBS containing 4% formaldehyde for 30 min. TUNEL assay was performed using the *in situ* cell Death Detection Kit (Roche Inc., USA) following the manufacturer’s instructions. Briefly, after TUNEL-staining according to the protocols, sections were subsequently incubated with 4′,6-diamidino-2-phenylindole (DAPI)-containing medium for 10 min. The TUNEL-positive cells were identified by a blind investigator via fluorescence microscopy (Leica Microsystems, Germany). Then, the data were analyzed by another blind investigator. We defined the apoptotic index as the average number of TUNEL-positive cells in six views of slices.

### Western Blot Analysis

After isoflurane exposure, the bilateral hippocampus tissues were dissected. Total protein from the hippocampus and neuronal cultures were harvested in RIPA lysis buffer (Beyotime Biotechnology, Shanghai, China), and mixed with phenylmethylsulfonyl fluoride protease inhibitor. Neurons were extracted from three wells in a culture plate. Separation and extraction of the cytoplasmic and nuclear proteins of hippocampal neurons were performed following the Tissue Protein Extraction Kit (Boster Biological Technology, Shanghai, China). Levels of protein were determined by using a BCA assay kit (Boster Biological Technology). Equal amounts of protein were subjected to electrophoresis via 10% sodium dodecyl sulfate-polyacrylamide gel and then transferred to 0.45-μm polyvinylidene difluoride (PVDF) membranes. After blocking with 5% defatted milk powder in TBST, membranes were incubated overnight at 4°C with primary antibodies: mouse anti-β-actin (1:500; Boster Biological Technology), rabbit anti-Cdh1 (1:1000; Abcam), rabbit anti-Skp2 (1:500; ABclonal), rabbit anti-cyclin B1 (1:1000; ABclonal), rabbit anti-SnoN (1:1000; Abcam), rabbit anti-Bax (1:1000; Cell Signaling Technology, Danvers, MA, USA), rabbit anti-Bcl-2 (1:1000; Cell Signaling Technology), rabbit anti-total Caspase-3 (1:1000; Cell Signaling Technology), or rabbit anti-cleaved-Caspase-3 (1:1000; Cell Signaling Technology). Membranes were rinsed with TBST, incubated with a horseradish peroxidase-conjugated secondary antibody (1:5000; Boster Biological Technology) for 1.5 h at room temperature. After rinsing with TBST three times, bands were detected with an enhanced chemiluminescence kit (Thermo Scientific, Waltham, MA, USA). Signals were digitally scanned with a Chemi-Doc XRS imaging system (Bio-Rad, Hercules, CA, USA). The relative protein levels were normalized to that of the housekeeping gene, β-actin.

### Quantitative Real-Time PCR

The levels of Cdh1 mRNA were examined via SYBR green-based quantitative RT-PCR. Total RNA was isolated from the rat hippocampi using Trizol reagent (Invitrogen, Carlsbad, CA, USA) according to the previous manufacturer’s instructions. The reaction solution was composed of cDNA template, forward/reverse primers, and SYBR green I Master Mix (Invitrogen, Carlsbad, CA, USA). The primers used to amplify Cdh1 and β-actin were as follows: *Cdh1*: forward: 5′-GAACCGCAAAGCCAAGGAT-3′, reverse: 5′-CTTGTGCTCTGGGGTGGAT-3′; β-actin: forward: 5′-CACGATGGAGGGGCCGGACTCATC-3′, reverse: 5′-TAAAGACCTCTATGCCAACACAGT-3′. Reverse transcription and real-time reverse transcription PCRs were performed in triplicate. Fold-change was calculated using the ΔΔCT method to estimate the amount of target mRNA.

### Open-Field Test

The open-field test was performed 21 days after isoflurane-treatment according to previously described protocols (Wang et al., 2012). On P28, rats (*n* = 10 for each group) were placed individually in a black box for 30 s in the dark for habituation. Then, each rat was allowed to explore from the center of the box for 10 min in dark. During the procedure, the behavior of the rat pups was recorded by using a video tracking system placed right above, which could automatically measure the distance traveled and the amount of time spent in the center area. When each test was completed, the inner wall of the chamber was wiped with 95% ethyl alcohol to prevent the influence of olfactory cues.

### Morris Water Maze Test

The morris water maze (MWM) test was performed 26 days after isoflurane-treatment as previously described, with some modifications (Ju et al., 2015). Rats (*n* = 10 for each group) were subjected to four trials per day over five consecutive days to evaluate the rats’ spatial memory between distant cues and the escape platform from P31 to P36. A circular platform (submerged 1.5 cm, not visible) was placed at one of the four quadrants (named the target quadrant [T]), and the remaining quadrants were defined by the relative location to the T quadrant: opposite (O), left (L) and right (R) quadrants. The release position was randomly predetermined. Each rat was given 60 s to locate the hidden platform. If the rat failed to find the platform within 60 s, it would be manually guided to the platform and allowed to stay on it for 15 s. The time to reach the platform could reflect the function of spatial learning and was named latency. The swimming paths were recorded by a digital video camera placed right above the tank. Immediately after the last training session (P36), the platform was removed, and a 60-s-probe trial was performed. Rats started to swim in the same quadrant facing the pool wall. The number of times the rats crossed the area where the platform was located in the target quadrant and the swimming time in each quadrant were recorded by a digital video camera positioned right above the center of the water maze. The recording data were further analyzed by using the DigBehav System (Jiliang Software Co., Shanghai, China).

### Statistical Analysis

All data are presented as mean ± standard error of the mean (SEM). The SPSS software (Version 17.0; SPSS Inc., Chicago, IL, USA) was used for statistical analysis. One-way analysis of variance (ANOVA), followed by Turkey honest significant difference (HSD) test and student’s *t*-test were used as appropriate for comparisons between different groups. Group comparisons in the spatial training sessions of the MWM test were analyzed by two-way repeated ANOVA, in which “Days of testing” was treated as the “within-subjects” factor and “Groups” was used as the “between-subjects” factor. Values of *P* < 0.05 were considered statistically significant.

## Results

### Exposure of Postnatal 7-Day Rat Pups to Isoflurane Leads to Cdh1 Downregulation and Neuronal Apoptosis in the Hippocampus

**Figure 1:**
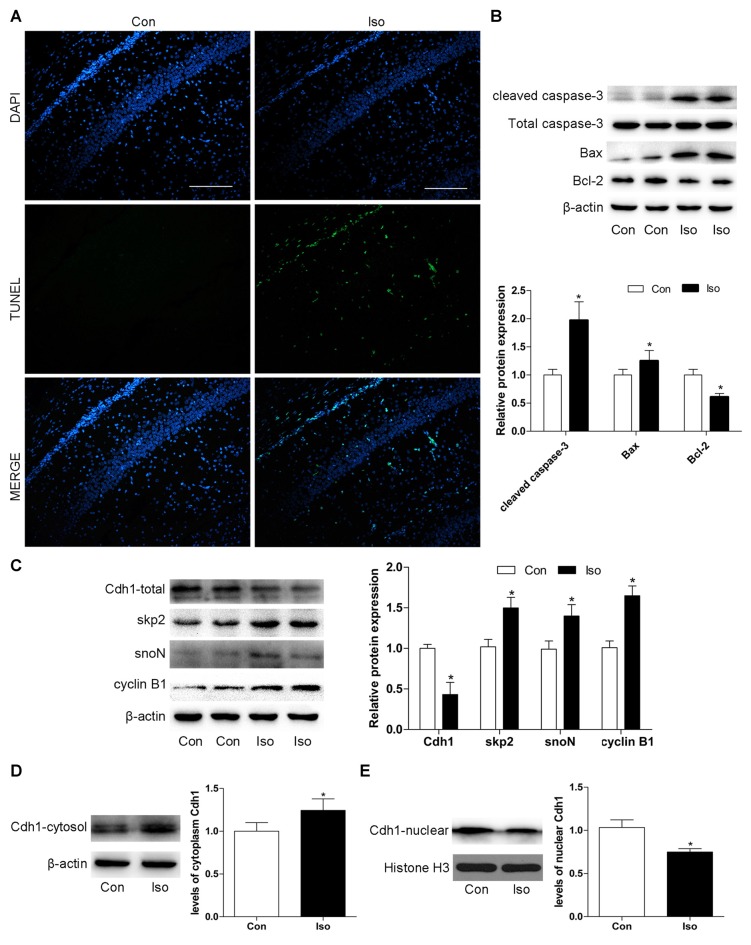
Exposure to isoflurane downregulates Cdh1 activity and induces neuronal apoptosis in the hippocampus of postnatal 7-day rat pups. Anesthesia was induced by placing rats at postnatal day 7 (P7) in an anesthetizing chamber prefilled with 30% oxygen (O_2_) and 70% nitrogen (N_2_) in the absence or presence of 2% isoflurane for 6 h. **(A)** After isoflurane exposure, apoptosis of hippocampal neurons was assessed by terminal deoxynucleotidyl transferase dutp nick end labeling (TUNEL)-assay. TUNEL-positive cells are labeled in green; blue, nuclei stained with 4′,6-diamidino-2-phenylindole (DAPI). Scale bar = 200 μm. **(B,C)** After isoflurane-treatment, rat hippocampal tissues were collected to assess the expression of apoptosis-related proteins (cleaved caspase-3, Bax, and Bcl-2), total Cdh1 and several downstream substrates (skp2, snoN and cyclin B1) by western blotting. Quantitative data for the western blot (*n* = 3 for each group) are shown by histograms. β-actin was set as an internal reference, and the fold change for density in the control group was used for quantification. **(D,E)** Representative levels of Cdh1 in the cytosol and nucleus. The histogram represents the quantitative analysis (*n* = 3 in each group). Data are expressed as mean ± standard error of the mean (SEM). *P < 0.05 vs. control group.

First, we assessed whether isoflurane at the concentration used in our experiment could induce neuronal apoptosis in the developing rats. As shown in Figure 1A, the number of TUNEL-positive cells significantly increased in the isoflurane group compared to that in the control group in the hippocampal CA1 region after anesthesia with 2% isoflurane for 6 h. Consistently, western blotting showed that isoflurane-treatment lead to a significant elevation of pro-apoptotic proteins (cleaved caspase-3 and Bax), while the expression of the anti-apoptotic protein Bcl-2 decreased after anesthesia (*P* = 0.006 for cleaved caspase-3, *P* = 0.017 for Bax and *P* = 0.004 for Bcl-2; Figure 1B). Additionally, isoflurane reduced the total level of Cdh1 in the developing hippocampus, as assessed by western blotting (*P* = 0.018, Figure 1C). We further analyzed Cdh1 activity according to subcellular localization. The results showed that the amount of Cdh1 in the cytosol was significantly higher in the isoflurane group than in the control group (*P* = 0.021, Figure 1D), while the level of Cdh1 in the nucleus was reduced after isoflurane-treatment (*P* = 0.032, Figure 1E). These results suggest the translocation of Cdh1 from the nucleus to the cytosol during isoflurane-treatment. In addition, together with the downregulation of the total level of Cdh1, several downstream substrates (skp2, snoN and cyclin B1) were accumulated, indicating inactivity of Cdh1 (*P* < 0.05, Figure 1C).

### Cdh1 Is Downregulated during Isoflurane-Induced Neuronal Apoptosis *in Vitro*

**Figure 2:**
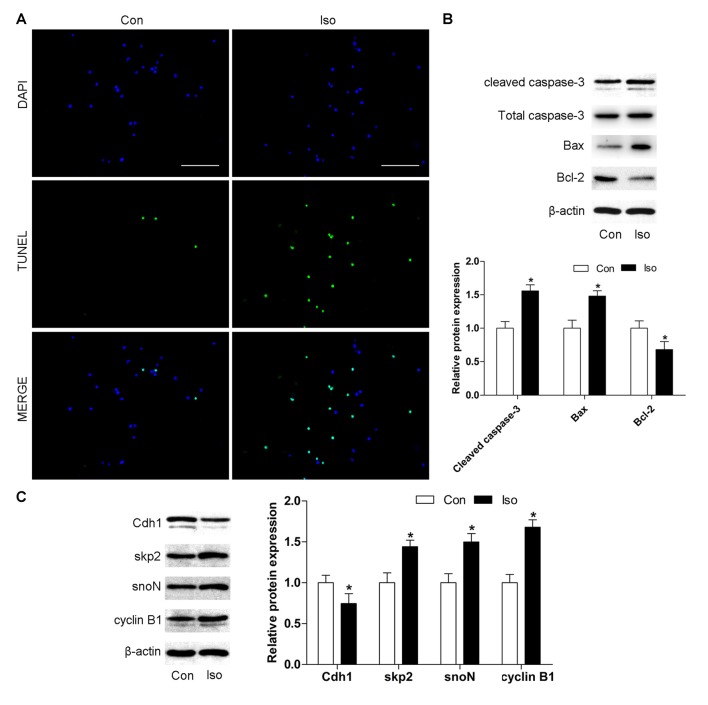
Cdh1 is downregulated during isoflurane-induced neuronal apoptosis *in vitro*. Cultured neurons were treated with 95% O_2_ and 5% carbon dioxide (CO_2_) in the absence or presence of 2% isoflurane for 6 h on day 7 *in vitro* (7 DIV). **(A)** Representative images of cultured neurons are shown after incubation with TUNEL reaction solution. Cells labeled in green are positive for apoptosis; blue, nucleus. Scale bar = 100 μm. **(B,C)** The level of pro-apoptotic proteins (cleaved caspase-3 and Bax) and anti-apoptotic protein Bcl-2 was detected by western blotting. In addition, Cdh1 and its downstream substrates (skp2, snoN and cyclin B1) were also assessed by western blot analysis. Quantification of these proteins is shown. Data are presented as mean ± SEM. *P < 0.05 vs. control group.

To further confirm the effects of isoflurane on neuronal apoptosis and Cdh1 activity, cultures of hippocampal neurons were exposed to isoflurane (2%, 6 h). Consistent with the results obtained *in vivo*, isoflurane-treatment alone led to significant neuronal cell death (Figure 2A). Western blot indicated an increase in the expression of cleaved caspase-3 and Bax proteins and a decreased level of Bcl-2 protein in the isoflurane-treatment group compared with the control group (*P* < 0.05, Figure 2B). Furthermore, Cdh1 level was decreased after exposure to isoflurane (*P* = 0.039), and the expression of downstream substrates of Cdh1 (skp2, snoN and cyclin B1) was enhanced in the isoflurane group compared with the control group (*P* < 0.05, Figure 2C). Altogether, these data further indicated that downregulation of Cdh1 activity is correlated with isoflurane-induced neuronal apoptosis.

### Lentivirus-Mediated Cdh1 Over-expression Reduces Isoflurane-Induced Neuronal Apoptosis *in Vitro*

**Figure 3:**
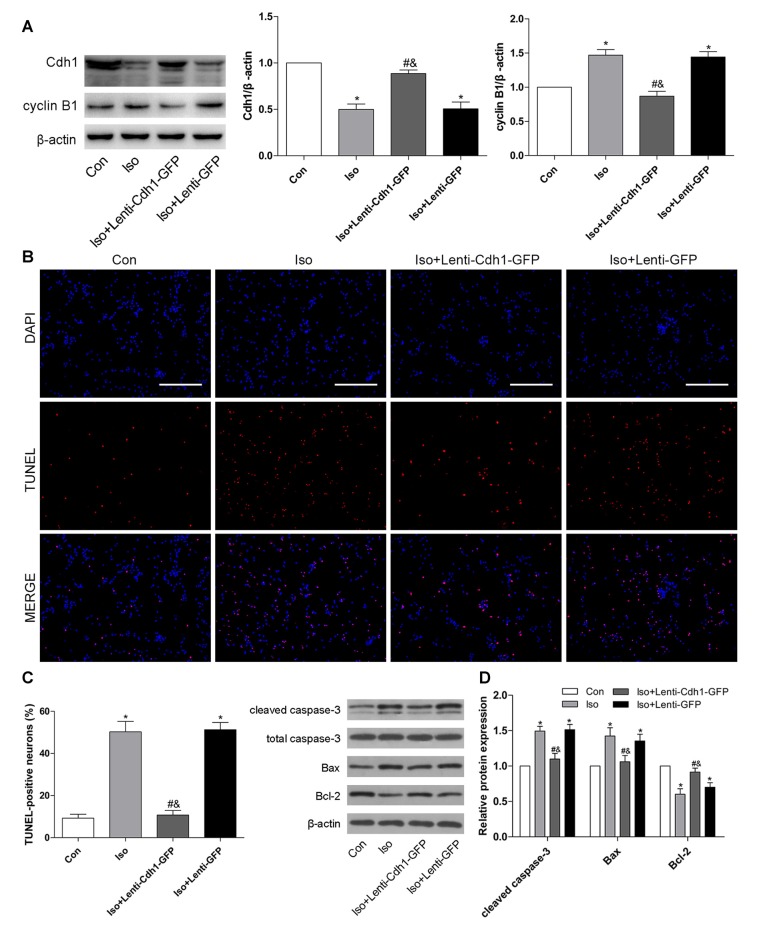
Lentivirus-mediated Cdh1 overexpression reduces isoflurane-induced neuronal apoptosis *in vitro*. Four groups were included in this experiment: Con, Iso, Iso + Lenti-Cdh1-green fluorescent protein (GFP), and Iso + Lenti-GFP groups. Cdh1-expressing lentivirus in the Iso + Lenti-Cdh1-GFP group and control lentivirus in the Iso + Lenti-GFP group were transfected to cultured neurons on 4 DIV for 10 h before isoflurane exposure (2%, 6 h) on 7 DIV. **(A)** Representative bands for the expression of Cdh1 and its downstream substrate cyclin B1 in the developing hippocampus after isoflurane anesthesia. The histograms represent the density values of Cdh1 and cyclin B1 separately. **(B)** Neuronal cell death was detected by TUNEL assay. TUNEL-positive cells are labeled in red, and the nucleus is stained blue. Scale bar = 200 μm. **(C)** The lower panel shows percentages of TUNEL-positive cells in each group. **(D)** After lentivirus pretreatment and isoflurane anesthesia, the expression of apoptotic proteins was analyzed by western blotting. Histograms show the quantification of each protein. *P < 0.05 vs. control group, ^#^*P* < 0.05 vs. Iso group, ^&^*P* < 0.05 vs. Iso + Lenti-GFP group.

To test whether Cdh1 upregulation would protect against isoflurane-induced developmental neurotoxicity, a lentivirus encoding Cdh1 was administered before isoflurane-treatment. For *in vitro* experiments, we chose an MOI of 10 as the optimal MOI to infect neurons on 4 DIV for 10 h; cells were treated with isoflurane 72 h after transfection. The expression of Cdh1 was effectively upregulated by Cdh1-encoding lentivirus in the Iso + Lenti-Cdh1-GFP group compared with the Iso group, while the accumulation of cyclin B1 was significantly reduced at the same time (*P* = 0.003 for Cdh1 and *P* < 0.05 for cyclin B1, lane 3, Figure 3A). Additionally, the number of TUNEL-positive cells was significantly decreased in the Iso + Lenti-Cdh1-GFP group, while there was no statistical difference in the number of apoptotic neurons between the Iso and Iso + Lenti-GFP groups (Iso + Lenti-Cdh1-GFP and Iso + Lenti-GFP group vs. Iso group, *P* < 0.001 and *P* = 0.638, Figures 3B,C). In addition, the activation of cleaved caspase-3 and Bax in the Iso group was abolished after transfection of the Cdh1-encoding lentivirus in the Iso + Lenti-Cdh1-GFP group. Moreover, Bcl-2 expression was enhanced in the Iso + Lenti-Cdh1-GFP group, while it was reduced in the Iso group (*P* < 0.05, lane 3, Figure 3D).

### Bilateral Intra-Hippocampal Injection of Cdh1-Encoding Lentivirus Decreases Isoflurane-Induced Neuronal Apoptosis in the Developing Hippocampus

**Figure 4:**
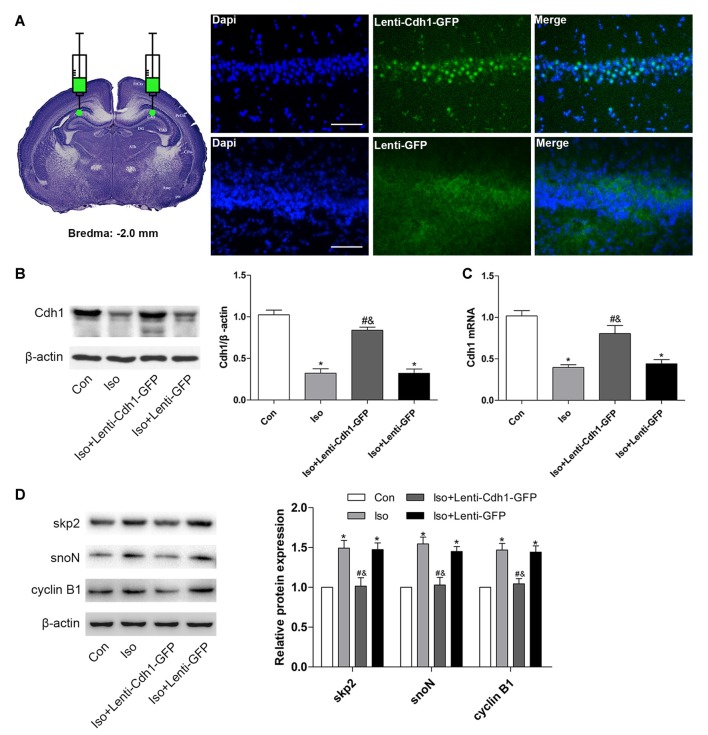
Cdh1 overexpression in the hippocampus in developing rats after bilateral intra-hippocampal injection with Cdh1-encoding lentivirus. Cdh1-expressing lentivirus (in the Iso + Lenti-Cdh1-GFP group) and control lentivirus (in the Iso + Lenti-GFP group) were injected into the bilateral hippocampus of rat pups at P3 before isoflurane inhalation on P7. **(A)** The picture demonstrates that intra-hippocampal injection of the lentivirus was administered according to the coordinates: the Bregma as zero; anterior, −2.0 mm; lateral, ±1.6 mm; dorsal, −2.5 mm. GFP-positive signals were mainly detected in the hippocampus and surrounding area after 3 days. **(B)** Western blotting was used to evaluate the expression of Cdh1 in the control and isoflurane-treatment groups (lanes 2–4). The histogram represents the relative levels of Cdh1 (*n* = 3 in each group). **(C)** The histogram shows the mRNA expression of Cdh1 after lentivirus-pretreatment and isoflurane-treatment by real-time PCR (*n* = 3 in each group). **(D)** The expression of Cdh1 substrates (skp2, snoN and cyclin B1) were analyzed by western blotting (*n* = 3 in each group). Values are presented as mean ± SEM. *P < 0.05 vs. control group, ^#^*P* < 0.05 vs. Iso group, ^&^*P* < 0.05 vs. Iso + Lenti-GFP group.

**Figure 5:**
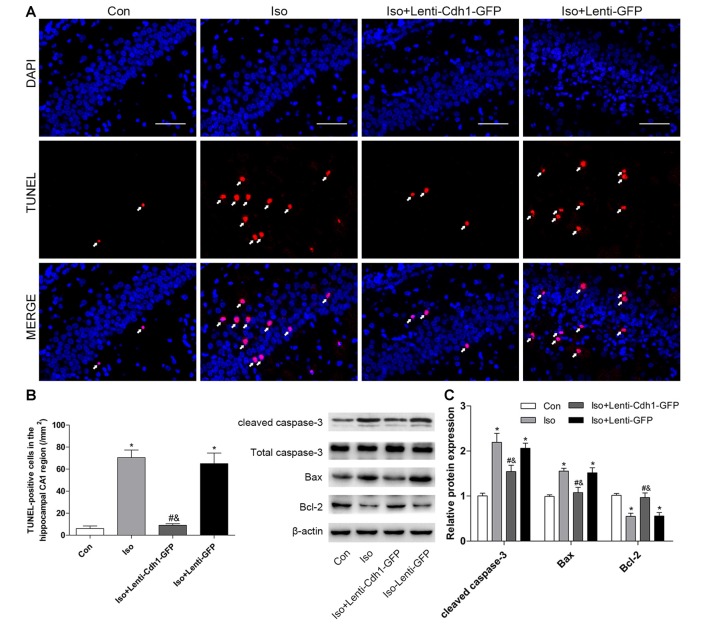
Bilateral intra-hippocampal injection with Cdh1-encoding lentivirus decreases isoflurane-induced neuronal apoptosis in the developing hippocampus. **(A)** Representative images of TUNEL-assay in the hippocampal CA1 region are shown. Red color indicates TUNEL-positive cells; blue indicates the nucleus. Scale bar = 50 μm. **(B)** The lower histogram shows the number of TUNEL-positive cells in the hippocampal CA1 region (*n* = 6 in each group). **(C)** The western blot bands (left) and quantitative data (right) show the effect of the lentivirus and isoflurane-treatment on the expression of cleaved caspase-3, Bax and Bcl-2 in the developing brain in different groups (*n* = 3 in each group). *P < 0.05 vs. control group, ^#^*P* < 0.05 vs. Iso group, ^&^*P* < 0.05 vs. Iso + Lenti-GFP group.

To further confirm the effects of Cdh1 overexpression on isoflurane-induced neuronal apoptosis, the lentivirus was stereotactically injected into bilateral CA1 of the hippocampus in rat pups. The expression of GFP was detected by immunofluorescence microscopy. The results showed that GFP was mainly expressed in the cytoplasm in the Iso + Lenti-GFP group, while it was expressed in the nucleus in the Iso + Lenti-Cdh1-GFP group, as GFP was fused to Cdh1 in the vector (Figure 4A). Western blotting and real-time PCR showed that Cdh1 expression was enhanced in the Iso + Lenti-Cdh1-GFP group compared with the Iso and Iso + Lenti-GFP group (*P* < 0.05, Figures 4B,C). The upregulation of downstream substrates of Cdh1 induced by isoflurane were reversed (*P* < 0.05, Figure 4D; lane 3). The number of TUNEL-positive cells in the hippocampal CA1 area was decreased by Cdh1 overexpression in the Iso + Lenti-Cdh1-GFP group (Iso + Lenti-Cdh1-GFP group vs. Iso group, *P* < 0.001; Iso vs. control group, *P* < 0.001; Figures 5A,B). Quantification of the western blot revealed that the administration of Cdh1-encoding lentivirus expectedly decreased the expression of pro-apoptotic proteins (cleaved caspase-3 and Bax) and rescued the anti-apoptotic protein Bcl-2 protein level (*P* < 0.05, lane 3, Figure 5C). Together, these results suggest that Cdh1 overexpression protected hippocampal neurons from apoptosis induced by isoflurane anesthesia in the developing brain.

### Cdh1 Overexpression Attenuates Isoflurane-Induced Long-Term Cognitive Deficits

**Figure 6:**
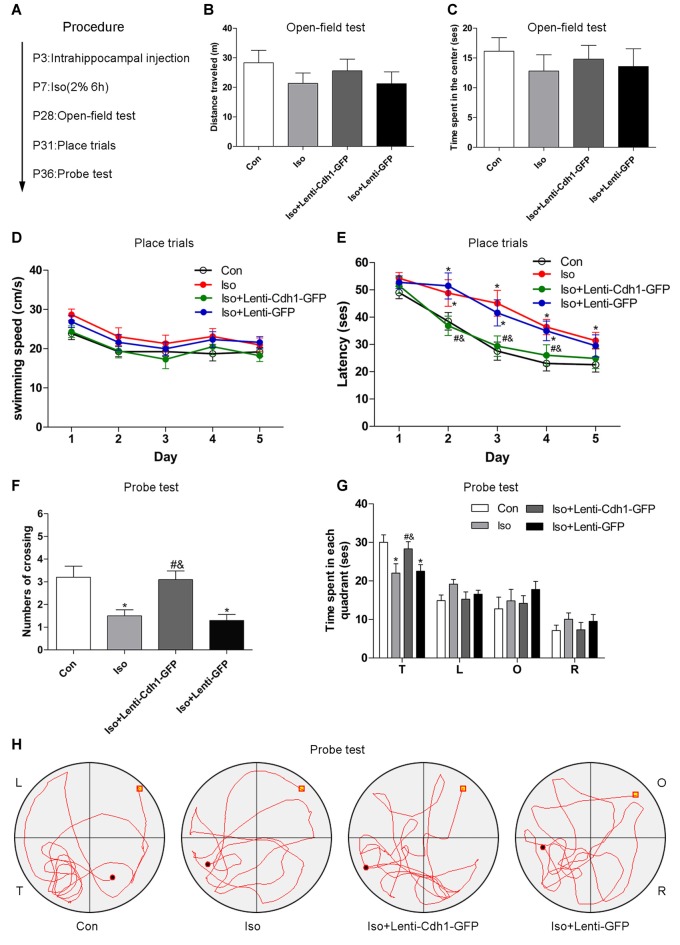
Cdh1 overexpression attenuates isoflurane-induced long-term cognitive deficits. **(A)** The experimental protocol of the behavioral test (*n* = 10 for each group). **(B,C)** Total distance traveled and time spent in the center was assessed in the open field test. No significance was observed among the four groups. **(D,E)** Swimming speed and time to find the submerged platform in different groups were measured during the spatial training session of the morris water maze (MWM) test. **(F)** The number of crossings of each group was calculated in the probe trial of the MWM test. **(G,H)** Time spent in all four quadrants and representative swimming trajectory of each group is shown in the probe trial of the MWM test. The behavior of rats in the control group was used as a standard. Values are presented as mean ± SEM. *P < 0.05 vs. control group, ^#^*P* < 0.05 vs. Iso group, ^&^*P* < 0.05 vs. Iso + Lenti-GFP group.

To investigate whether Cdh1 overexpression could protect against isoflurane-induced cognitive impairment in developing rats, a behavioral study was conducted (Figure 6A).

Compared with the control group, rats did not show abnormal locomotor and anxiety-like behavior in the isoflurane group, assessed on the basis of the distances traveled by the rats (control vs. Iso group, *P* = 0.226, Figure 6B) and the time spent in the center (control vs. isoflurane, *P* = 0.379, Figure 6C) during the open-field test, indicating that there were no physical and emotional depression between the experimental and control rats in terms of locomotor activity.

Hippocampal pyramidal neurons in the CA1 area are essential for spatial learning and memory. In the place trials, no difference was observed in terms of swimming speeds (control vs. Iso group: *P* > 0.05 from day 1 to day 5, Figure 6D), consistent with the results obtained for locomotor activity. However, rats in the isoflurane group and Iso + Lenti-GFP group apparently spent more time in finding the submerged platform than the control group rats from trial day 2 to day 4 (control vs. Iso and Iso + Lenti-GFP group: *P* = 0.048 and 0.011, second trial; *P* = 0.009 and 0.031, third trial; *P* = 0.011 and 0.009, fourth trial; Figure 6E). The rats had shorter escape latency in the Iso + Lenti-Cdh1-GFP group than in the Iso group or Iso + Lenti-GFP group from trial day 2 to day 4 (*P* = 0.038 and 0.036, second trial; *P* = 0.018 and 0.047, third trial; *P* = 0.007 and 0.006, fourth trial; Figure 6E). In the probe test, rats from the Iso and Iso + Lenti-GFP groups showed a reduction in the number of platform crossing and time spent in the target quadrant compared with the control group (*P* = 0.002 and 0.001, respectively, Figure 6F). In addition, animals in the Iso + Lenti-Cdh1-GFP group exhibited increased number of crossing and time spent in the target quadrant compared to rats from the Iso and Iso + Lenti-GFP groups (*P* = 0.003 and 0.001 for crossing; *P* = 0.04 and 0.046 for time, Figures 6F,G). Furthermore, the typical swimming paths of each group are shown in Figure 6H. Collectively, these data suggested that Cdh1 overexpression alleviated the learning and memory deficits caused by isoflurane exposure in the developing rats, but did not influence locomotor activity.

## Discussion

Our findings demonstrated that exposure to 2% isoflurane for 6 h induces neuroapoptosis and significantly reduces Cdh1 expression, coinciding with the accumulation of several of its downstream substrates (skp2, snoN and cyclin B1). Additionally, Cdh1 overexpression attenuated the neuroapoptosis induced by isoflurane exposure. Furthermore, through intra-hippocampal injection of lentivirus encoding Cdh1, we observed that overexpressing Cdh1 in the developing hippocampus ameliorated the learning and memory impairments caused by isoflurane anesthesia. To our knowledge, this is the first study to investigate the role of APC-Cdh1 in isoflurane neurotoxicity, and our results suggest that Cdh1 is associated with isoflurane-induced neurotoxicity.

Generally, the immature rat brain is most vulnerable to neuronal apoptosis induced by anesthesia during the process of synaptogenesis (Jevtovic-Todorovic et al., 2013). High concentration or long duration of anesthetics exposure could lead to significant neurotoxicity. The minimum alveolar concentration (MAC) of isoflurane was 2.7% in P7 Sprague-Dawley rats (Istaphanous et al., 2011). In this study, we employed a moderate dose of isoflurane (2%), which is within the clinically used dosage during pediatric anesthesia (Cameron et al., 1984). Short exposures to a low concentration of isoflurane did not cause cell damage (Zhao et al., 2013). A single anesthesia exposure before the age of 36 months among healthy children did not result in differences in IQ scores in later childhood, compared with healthy siblings with no anesthesia exposure (Sun et al., 2016). Therefore, we determined 6 h as the exposure time based on previous studies (Lei et al., 2012). Consistent with a previous study (Zhang et al., 2010), our results demonstrated that isoflurane exposure increases the number of apoptotic cells in the hippocampal CA1 area and in cultured neurons. In addition, disruption of the balance between anti-apoptotic protein Bcl-2 and pro-apoptotic protein Bax occurs, which ultimately stimulates caspase-3 and neuronal apoptosis.

Different methods can be used to evaluate the activity of APC. APC can be activated by combination with Cdh1. APC/C-Cdh1 displayed high ubiquitination activity in the purified brain. In the central nervous system, Cdh1 is highly expressed in post-mitotic neurons, and Cdh1 downregulation by RNA interference can reduce APC/C-Cdh1 activity (Konishi et al., 2004; Almeida et al., 2005). Another indirect method is to evaluate the level of APC substrates (Gieffers et al., 1999). In addition, Cdh1 phosphorylation and the following nuclear export lead to inactivity of APC/C-Cdh1 (Jaquenoud et al., 2002). In our study, Cdh1 total protein level was decreased after isoflurane exposure. Meanwhile, the expression of downstream substrates of APC/C-Cdh1 (skp2, snoN and cyclin B1) increased after isoflurane anesthesia, indicating that the ubiquitination activity of APC/C-Cdh1 was inhibited. Furthermore, Cdh1 was highly expressed in the nuclear of hippocampal neurons in the control group, whereas, in the isoflurane group, the expression of Cdh1 in the nucleus decreased, while that in the cytoplasm increased. These results demonstrated that the activity of APC/C-Cdh1 was downregulated during isoflurane-induced neuronal apoptosis in the developing brain. We only studied Cdh1 activity by measuring its location and the change of substrates, a direct method which correlates Cdh1 activity with the observed Cdh1 effect might be better for this study. Further studies are needed to address this point.

To further refine the role of Cdh1 in developmental isoflurane neurotoxicity, a lentivirus encoding Cdh1 was applied in our study. Lentiviral vectors can mediate transfer into any neuronal cell types and allow sustained expression without immune response in the developing mouse brain and primary neuron culture (Sun and Gan, 2011; Artegiani and Calegari, 2013). We previously constructed the lentivirus and successfully overexpressed Cdh1 in the nervous system (Lv et al., 2015; Hu et al., 2016). In the current study, we employed a recombinant Cdh1-encoding lentiviral vector to overexpress Cdh1 in the hippocampal neurons 3 days before isoflurane-treatment. GFP-positive signal was detected in the cytoplasm and nucleus of hippocampal neurons in developing rats. Western blot analysis and real-time PCR indicated that Cdh1 was overexpressed, indicating the effective transfection of Cdh1-encoding lentivirus.

Growing evidence has demonstrated that impaired function of APC/C-Cdh1 and accumulation of its substrates are involved in neurodegenerative diseases (Fuchsberger et al., 2017). APC/C-Cdh1 is involved in excitotoxicity, oxidative stress, and ectopic cell-cycle re-entry. APC/C-Cdh1 can cause aberrant stabilization and accumulation of cyclin B1 during excitotoxic damage (Maestre et al., 2008). Stabilized cyclin B1 binds and activates Cdk1, and accumulation of cyclin B1-Cdk1 complex continues to phosphorylate the anti-apoptotic protein Bcl-xL and leads to neuronal death (Veas-Pérez de Tudela et al., 2015a). Bax, a member of the Bcl-2 family, can also be regulated by APC-Cdh1 through degradation of the Bax activation enhancer (modulator of apoptosis protein 1; Lee et al., 2009; Huang et al., 2012). Isoflurane induces neuron apoptosis through the Bcl-2 family protein-mediated mitochondrial pathway of apoptosis (Zhang et al., 2010). Therefore, we assessed changes in cyclin B1 and Bcl-2 family proteins expression after Cdh1 overexpression and isoflurane anesthesia. Overexpression of Cdh1 not only decreased the accumulation of cyclin B1 *in vitro*, but also diminished the number of TUNEL-positive cells after isoflurane anesthesia. Consistently, the imbalance of Bcl-2 family proteins tended toward stabilization. Moreover, the caspase-3 activation induced by isoflurane was inhibited by Cdh1 overexpression. Therefore, Cdh1 overexpression protected hippocampal neurons against isoflurane-induced apoptosis.

It has been demonstrated that progressive neuronal death is a consequence of aberrant re-entry into the cell cycle of post-mitotic neurons in many neurodegenerative diseases (Herrup, 2013; Veas-Pérez de Tudela et al., 2015b). Inhibition of Cdh1 has been reported to mediate aberrant cell cycle entry of post-mitotic neurons, leading to apoptotic cell death. Cdh1 depletion in post-mitotic neurons can trigger cyclin B1-mediated entry into S-phase (Almeida et al., 2005). Moreover, Cdk5 phosphorylates Cdh1 on at least three phosphorylation sites, subsequently leading to cyclin B1 stabilization (Maestre et al., 2008). Notably, a recent study reported that neonatal isoflurane exposure caused aberrant Cdk5 stabilization (Wang et al., 2014). Therefore, the upstream partner of Cdh1 involved in isoflurane-induced neuronal apoptosis could be Cdk5. The downstream partner of Cdh1 could stabilize cyclin B1 to avoid re-entry into the cell cycle, thereby inducing apoptotic cell death. Further studies are needed to determine the relationship of Cdk5 activity and Cdh1 subunit expression in developmental isoflurane cytotoxicity.

Neonatal exposure to isoflurane anesthesia in rats produces neurobehavioral defects persisting into adulthood (Jevtovic-Todorovic et al., 2003; Zhu et al., 2010; Coleman et al., 2017). Neuroapoptosis occurs in cognition-related brain regions (such as the hippocampi) and produces an adverse impact on the fundamental development of neuronal networks, which leads to long-term neurocognitive decline. Many studies have supported the role of hippocampal neuroapoptosis in cognitive dysfunction (Jevtovic-Todorovic et al., 2003; Boscolo et al., 2012; Wu et al., 2017). Similarly, our results indicated that Cdh1 overexpression not only reduced isoflurane-induced hippocampal neuroapoptosis, but also alleviated the learning and memory deficits induced by isoflurane in developing rats.

Besides neuroapoptosis, other factors contribute to cognitive deficits associated with isoflurane exposure during brain development. Some studies support the notion that hippocampal synaptic plasticity disturbance (Uchimoto et al., 2014) and developmental neurogenesis suppression (Jevtovic-Todorovic, 2011; Kang et al., 2017) play important role in cognitive impairment induced by postnatal anesthesia exposure. APC/C-Cdh1 is important in contributing to synaptic development and transmission. Previous studies have reported that removing neuronal Cdh1 early in the development resulted in impaired hippocampal long-term potentiation (LTP) and deficits in behavioral flexibility during the updating of memories (Pick et al., 2013). Furthermore, conditional knockout of Cdh1 profoundly impaired the induction of long-term depression (LTD) in CA1 hippocampal neurons (Huang et al., 2015). Deletion of Cdh1 disrupted synapse development in the cortex and hippocampus (Bobo-Jiménez et al., 2017). In addition, recent studies have demonstrated that functional APC/C-Cdh1 ubiquitin ligase is required for developmental neurogenesis (Delgado-Esteban et al., 2013). These results indicated that downregulation of Cdh1 may be associated with the molecular mechanism of synaptic plasticity disturbance and neurogenesis suppression induced by isoflurane. However, further studies are needed to clarify the involvement of synaptic plasticity in anesthetic neurotoxicity.

Many preclinical and clinical studies focused exclusively on cognitive function and learning disability as the primary outcomes after anesthesia exposure at a young age. We examined the effect of isoflurane on postnatal rats in terms of other functional outcomes such as locomotor and anxiety-like explorative activity. Similar to a clinical study in children (Sun et al., 2016), there was no statistical difference in motor/processing speed after a single anesthesia exposure. It was reported that children before 2 years old repeatedly exposed to anesthesia resulted in higher risk of attention-deficit/hyperactivity disorder (Sprung et al., 2012). Although our preliminary behavioral studies suggest alleviation of learning deficits and no alteration of anxiety by overexpression of Cdh1 in isoflurane-treated rats, more cohorts and tests are needed to confirm our observations reported here. Therefore, cautions should be taken to generalize the results until further data becomes available.

This study has some limitations. First, we detected the effect of anesthesia alone on cognitive deficits in developing brain. However, it is impossible to administer anesthetics to children without surgery or examination in clinical practices. In fact, a surgical procedure can increase cytokine levels, and alter gene expression and neural plasticity, compared with administration of anesthesia alone (Broad et al., 2017). Second, we did not consider the gender-related effects and used mixed gender rats in our experiments. Previous studies have indicated that isoflurane exposure in newborn rats induces long-term cognitive dysfunction in males but not females (Lee et al., 2014), but the effect of gender on anesthetic-induced developmental neurotoxicity remains poorly understood. Third, we performed DAPI + TUNEL to represent the apoptosis of neurons, the neuronal maker NeuN might be better to detect neuron cell death. In addition, we assume that the TUNEL-positive cells do not express Cdh1 but we are currently unable to provide prove for it.

In conclusion, this is the first study to investigate the role of the UPS in anesthesia-induced neurotoxicity and bring a new prospect for neuroprotection in developmental anesthesia. Our study demonstrates that Cdh1 is downregulated in isoflurane-induced neuroapoptosis, leading to long-term cognitive deficits in rats. Cdh1 overexpression can prevent isoflurane-induced developmental neurotoxicity.

## Author Contributions

KW, LL and WY conceived and designed the experiments. XL, KW, RH and BZ were responsible for performing experiments. RH, LL, LW and CZ contributed to acquiring and analyzing data. XL, KW and WY analyzed data. XL and WY interpreted data and complete the manuscript. Authors included in our article agreed with the final manuscript.

## Conflict of Interest Statement

The authors declare that the research was conducted in the absence of any commercial or financial relationships that could be construed as a potential conflict of interest.
